# Rapid metagenomics analysis of EMS vehicles for monitoring pathogen load using nanopore DNA sequencing

**DOI:** 10.1371/journal.pone.0219961

**Published:** 2019-07-24

**Authors:** Taylor Sheahan, Rhys Hakstol, Senthilkumar Kailasam, Graeme D. Glaister, Andrew J. Hudson, Hans-Joachim Wieden

**Affiliations:** Alberta RNA Research and Training Institute, Department of Chemistry and Biochemistry, University of Lethbridge, Lethbridge, Alberta, Canada; Wilfrid Laurier University, CANADA

## Abstract

Pathogen monitoring, detection and removal are essential to public health and outbreak management. Systems are in place for monitoring the microbial load of hospitals and public health facilities with strategies to mitigate pathogen spread. However, no such strategies are in place for ambulances, which are tasked with transporting at-risk individuals in immunocompromised states. As standard culturing techniques require a laboratory setting, and are time consuming and labour intensive, our approach was designed to be portable, inexpensive and easy to use based on the MinION third-generation sequencing platform from Oxford Nanopore Technologies. We developed a transferable sampling-to-analysis pipeline to characterize the microbial community in emergency medical service vehicles. Our approach identified over sixty-eight organisms in ambulances to the genera level, with a proportion of these being connected with health-care associated infections, such as *Clostridium spp*. and *Staphylococcus spp*. We also monitored the microbiome of different locations across three ambulances over time, and examined the dynamic community of microorganisms found in emergency medical service vehicles. Observed differences identified hot spots, which may require heightened monitoring and extensive cleaning. Through metagenomics analysis it is also possible to identify how microorganisms spread between patients and colonize an ambulance over time. The sequencing results aid in the development of practices to mitigate disease spread, while also providing a useful tool for outbreak prediction through ongoing analysis of the ambulance microbiome to identify new and emerging pathogens. Overall, this pipeline allows for the tracking and monitoring of pathogenic microorganisms of epidemiological interest, including those related to health-care associated infections.

## Introduction

The epidemic potential of disease causing microorganisms (pathogens) and the spread of antibiotic resistance continues to pose a serious threat to global health security [1]. Examples of such emergent threats include the bacterial meningitis epidemic in Sub-Saharan Africa [2] and the cholera outbreak in Haiti (2010) [3]. As a result, the World Health Organization (WHO) has acknowledged the need for a rapid pathogen detection platform to mitigate pathogen transmission and manage outbreaks in a reasonable timeframe [1]. Additionally, the rise of multidrug resistance in pathogens linked to health-care associated infections (HAIs), such as *Clostridium difficile* and *Staphylococcus aureus*, highlight the importance of pathogen identification and monitoring of antibiotic resistance genes to reduce their impact on disease spread and to combat the rise of antibiotic resistance [4, 5]. Within Canada, HAIs lead to death in more than 8,000 cases each year, indicating that HAIs are a serious public health concern [6]. For example, between 2008 and 2012 a reported 9% of patients infected with methicillin-resistant *Staphylococcus aureus* (MRSA) died [7]. The cost associated with increased morbidity and mortality stemming from HAIs is significant and must also be considered.

In response to the threat of HAIs and pathogen-associated disease, most hospitals have implemented pathogen monitoring and decontamination procedures to help prevent the spread and occurrence of HAIs, including standardized guidelines for hand hygiene and surface cleaning [8–11]. In contrast, no standardized evidence-based pathogen monitoring or detection process currently exists within the emergency medical services (EMS) sector, which plays a pivotal role in prehospital care. Ambulances, which frequently transfer injured, immunocompromised and often infectious patients between various public and hospital environments, are potential vehicles of HAI transmission [12]. The absence of a pathogen detection and reporting framework makes pathogen exposure risks difficult to evaluate and monitor. Furthermore, this absence hinders the identification of suitable strategies to mitigate pathogen transfer to patients, EMS personnel and the public.

Little is known about the epidemiological importance of EMS-patient contact on pathogen transmission between the community and hospitals, as well as the types of pathogens contained within ambulances. A limited number of studies from 1986 to 2016 have reported on the types of bacteria found in ground ambulances, but have not addressed the complete microbial environment or transmission of pathogens between the EMS, patients and community [12–25]. In these studies, swabs or air samples were taken and bacteria were identified by subsequent culturing under varied conditions depending on the pathogen of concern. This approach provides a biased view of the vehicle microbiome, focusing on specific types of bacteria and consequently neglecting slow-growing bacteria, unculturable bacteria, eukaryotes or viruses that are undetectable via cell culturing. Additionally, the dependence on a microbiology laboratory increases the time (>48 hours) and cost associated with the identification of microbes present. A review by Hudson *et al*. (2017) provides a more detailed summary of the literature on pathogen prevalence in EMS vehicles [26].

As an alternative to previous cell-culturing methods, a study by O’Hara and colleagues (2017) used a shotgun sequencing approach to profile the microbial community of ambulances within the United States [27]. Their results showed the identification of microbial species associated with HAIs, highlighting the use of metagenomic techniques to characterize the ambulance microbiome. Although promising as a pathogen identification tool, no platform has been developed to rapidly and portably monitor the dynamic microbial community of ambulances which changes drastically with patient turnover, emergency personal turnover and cleaning cycles.

In this study, we aim to develop a detection and monitoring platform with the potential for on-site and rapid sequencing utilizing the MinION Nanopore sequencing device from Oxford Nanopore Technologies (ONT). This device offers several key advantages to previous DNA sequencing platforms; it is relatively inexpensive, small, and portable providing the potential to perform DNA sequencing in remote locations [28–32]. The MinION device is also capable of producing ultra-long sequencing reads (>10,000 bp) [33], enabling easier *de novo* whole genome sequencing of bacteria [34–36], viruses [37–39] and eukaryotes [40–42]. While current versions of the MinION show lower read accuracy compared to more established sequencing platforms, such as Illumina MiSeq, Illumina HiSeq and PacBio RSII, similar resolution has still been achieved with this technology [43–45]. Additionally, Nanopore DNA sequencing is expected to improve with continued product development. The MinION device has already shown potential as a pathogen surveillance tool [37, 46, 47], including detection of Ebola virus transmission in remote Guinea West Africa [28], monitoring *Salmonella* outbreaks in hospitals [48], identifying antibiotic resistance markers in clinical settings [49–52], and for the real-time analysis of infected prosthetic devices [53].

We utilized the MinION sequencing technology to develop and demonstrate a complete workflow for characterizing microbial communities in ambulance vehicles. DNA samples recovered from surface swabs of three high-risk areas were obtained from three ambulances over a three-week period and subjected to partial 16S ribosomal RNA (rRNA) gene amplification and MinION DNA sequencing of amplicons for microbial identification. The 16S rRNA gene has been shown previously to be a useful molecular marker for bacterial identification, including for pathogens with clinical relevance [54]. This sequencing method and analysis pipeline demonstrates the utility of this technology for the rapid surveillance of pathogenic organisms in EMS vehicles. Additionally, we demonstrate the ability to identify important areas in the vehicle to monitor, inform best cleaning practices and suggest what role the EMS has on the spread of microorganisms and the ambulance microbiome in general. The developed technology is highly portable and scalable, which enables wide adoption by EMS in order to safeguard themselves and others from harmful microorganisms.

## Materials and methods

### Study design

**Fig 1:**
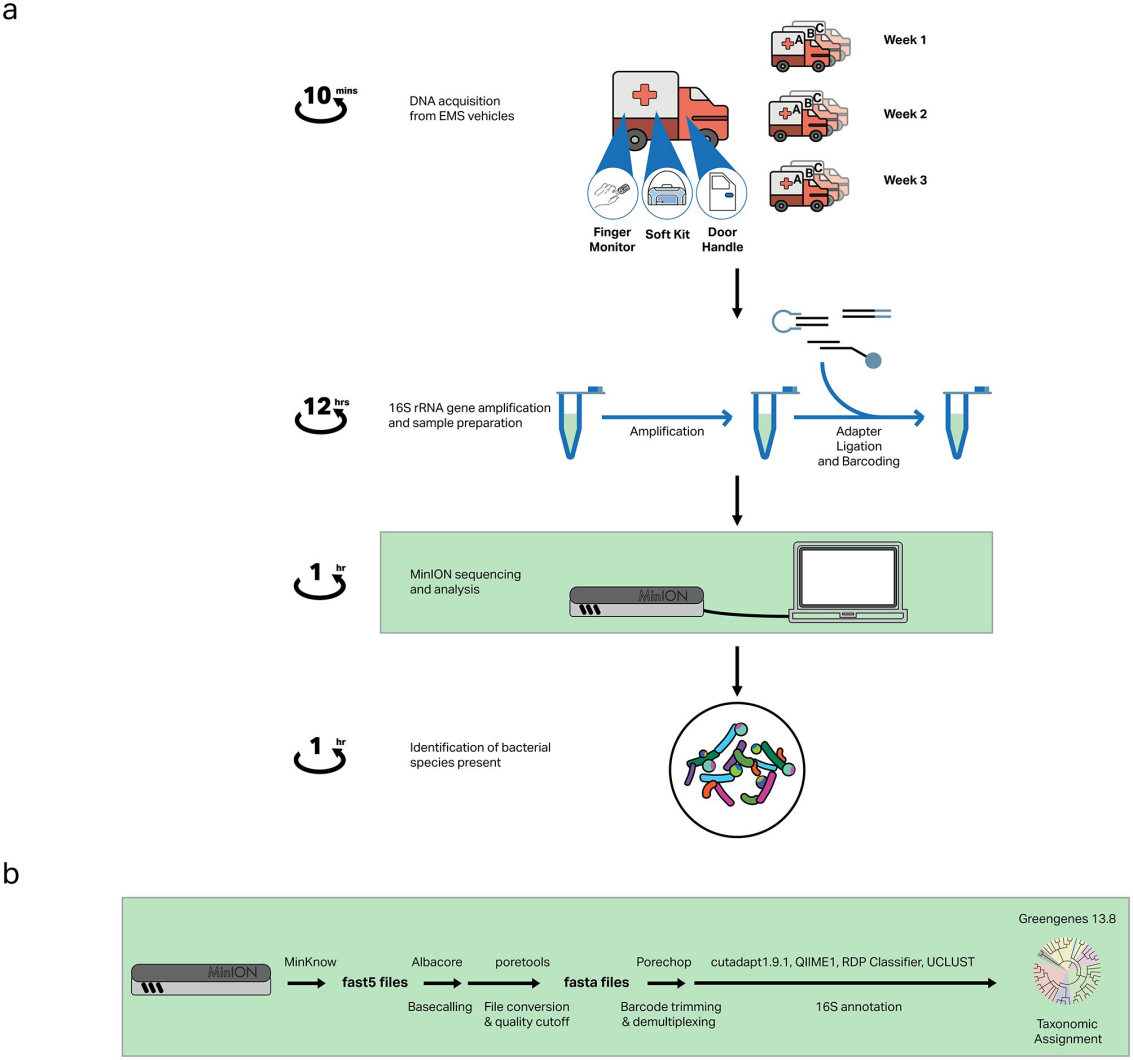
Nanopore sequencing workflow to rapidly identify bacteria in ambulances. (A) Samples are acquired from three locations in three vehicles over a three-week period, followed by DNA isolation, 16S rRNA gene amplification and sample preparation. The prepared library is then sequenced using the MinION and analyzed to identify bacterial species present. Taxonomic identification is possible within 24 hours from initial sample acquisition. (B) Bioinformatics analysis pipeline for generating taxonomic data.

To assess the variation of the microbial community over time and between EMS vehicles, three locations were sampled from three vehicles (Ambulance A, B and C) once a week over a three-week period. A sampling control was included to mimic the sampling procedure without physically touching any surface, providing a baseline measurement of bacteria present on the swab or introduced during processing. The sampling workflow (Fig 1A) was designed to coincide with standard cleaning protocols, beginning immediately after an extensive deep-clean, in which all surfaces were cleaned thoroughly, followed by routine cleaning over the three-week period.

Three high-risk contamination areas were chosen to be sampled: (i) SPO_2_ finger monitor, (ii) rear cab interior door handle and (iii) bottom of the soft kits. These locations were identified by taking into consideration concerns expressed by EMS workers, locations sampled in previous work [16, 18, 20, 21, 24], and our own observations from ride-alongs, as well as interviews with emergency personnel. The primary contact of each sample location varies, representing diverse sources of contamination and the potential for pathogen transmission to different locations. Specifically, the SPO_2_ finger monitor is frequently used and comes into direct skin contact with patients, providing specific information on *patient contamination*. The rear cab interior door handle is used by emergency personnel when transferring patients from the field into the vehicle and out of the vehicle to the hospital, providing information on *EMS worker contamination*. Lastly, the soft kits are transported on-site to each emergency call and are exposed to diverse environments, therefore it is representative of *environmental contamination*.

### Sampling procedure

Samples were acquired from each location under sterile conditions. A 4 cm^2^ area of each location was swiped using Grade I 15 mm diameter Whatman filter paper (GE Healthcare Life Sciences, Mississauga, ON, CAN) soaked in 70% ethanol. Samples were stored in 500 μL of 70% ethanol at -20°C until further processing. Sampling kits were prepared under sterile conditions prior to sampling. Nitrile gloves and sterile tweezers were used when handling filter paper to minimize external sample contamination. Each week an environmental control was included to identify bacteria introduced after sampling or during subsequent processing, where the filter paper was handled to mimic the sampling procedure but did not physically swipe a location.

### DNA extraction

To extract DNA from the filter paper, a protocol from [55] was adapted. Ethanol was evaporated using a rotary evaporator for 2 hours. 400 μL of alkaline lysis buffer (200 mM KOH, 50 mM DTT) was added to each filter paper and mixed by inversion. Samples were incubated on a heat block at 65°C for 10 min and centrifuged at 17 000 x g for 1 min. 300 μL of the sample was transferred to a new 1.5 mL micro centrifuge tube. 30 μL of 3M sodium acetate (pH 5.3), 5 μL of 0.5% linear polyacrylamide (LPA) carrier and 750 μL of cold 100% ethanol was added to each tube and vortexed. The samples were placed on ice for 10 min and centrifuged at 17 000 x g for 5 min. The resulting pellet was washed twice with 500 μL of 70% ethanol and re-suspended in 20 μL of filtered nuclease-free water.

### Bacterial 16S rRNA gene amplification

Specific universal primers, U341F (5' - CCTACGGGRSGCAGCAG - 3') and U1053R (5' - CTGACGRCRGCCATGC – 3’) [56], were used to amplify the hypervariable regions V4, V5 and V6 covering approximately 50% of the 16S rRNA gene. These regions were previously identified *in silico* as reliable and sensitive biomarkers for taxonomic classification and phylogenetic studies [57]. ~10 ng of environmental DNA was amplified via polymerase chain reaction (PCR) using Phusion High-Fidelity polymerase (Thermo Scientific, Waltham, MA, USA). The thermocycler conditions were set at 98°C for 2 min; 30 cycles of 98°C for 20 s, 64°C for 15 s and 72°C for 30 s; followed by 72°C for 2 min. Amplicons were confirmed using gel electrophoresis.

### Amplicon barcoding and library preparation

For each week of samples, barcoding kit 1 (Oxford Nanopore Technologies, Oxford, UK) and sequencing kit SQK-NSK007 (Oxford Nanopore Technologies) were used to generate the DNA library of pooled samples for sequencing. Less than 1,000 ng of purified amplicon DNA was end repaired and dA-tailed using the NEBNext Ultra II End-Repair/dA-tailing Module (NEB, Ipswich, MA, USA) incubating at 20°C for 5 min and 65°C for 5 min, followed by AMPure XP bead (Beckman Coulter Inc., Pasadena, CA, USA) cleanup per the manufacturer’s instructions. Approximately 10–30 ng/μL was recovered. 20 μL of Adaptor Mix (Oxford Nanopore Technologies, SQK-NSK007) was added to 30 μL of the dA-tailed DNA. From the dA-tailed/Adaptor Mix, 5 μL was removed, added to 5 μL of Blunt/TA ligase master mix (NEB) and incubated at room temperature for 10 min, followed by EZ-10 spin column purification (BioBasic). For a 50 μL PCR barcoding reaction, 1 μL of barcode from 1 to 12 (Oxford Nanopore Technologies, Barcoding Kit 1) was added to 24 μL of ligated product and amplified using Phusion High Fidelity polymerase (Thermo Scientific). PCR barcoding conditions were as follows: 98°C for 2 min; 25 cycles of 98°C for 15s, 64°C for 15s and 72°C for 1 min; followed by 72°C for 2 min. For each week of samples, the barcoded DNA was pooled together in the appropriate ratios to prepare 1 μg of DNA in 45 μL of nuclease-free water. The pooled barcoded DNA was end-repaired and dA-tailed using the NEBNext Ultra II End-Repair/dA-tailing Module (NEB) at 20°C for 5 minutes and 65°C for 5 min, followed by AMPure XP bead (Beckman Coulter Inc.) cleanup following the aforementioned protocol. 8 μL of nuclease free water, 10 μL of Adaptor mix, 2 μL of Hairpin HP Adaptor (HPA), and 50 μL of Blunt/TA ligase master mix was added to the dA-tailed pooled DNA and incubated at room temperature for 10 min. Then 1 μL of hairpin tether (HPT) was added and incubated at room temperature for 10 min. The adapted, tethered DNA was purified using MyOne C1 beads (Thermo Scientific) and eluted in 25 μL of elution buffer, while incubating at 37°C for 10 min. 5 μL of internal control DNA (DNA CS) was added to the sampling control for each sequencing preparation prior to barcoding, and to the pooled samples after barcoding to assess sequencing accuracy. An additional sample containing *Pseudomonas aeruginosa* genomic DNA was included as a second internal control to validate amplification accuracy.

### Nanopore sequencing

An R9.4 SpotON MinION flowcell was used and primed with Nanopore Priming Buffer in a stepwise fashion, loading 800μL, and after 10 min, loading an additional 200μL. Platform quality control (QC) was performed prior to loading of the priming buffer to ensure proper functioning of the MinION device. The library was then loaded onto the flowcell in a dropwise fashion, allowing each drop to enter into the flowcell before loading the next. The MinION was connected to the analysis computer and run using MinKNOW Version 1.4.2 using the SQK-LSK208 protocol for 2D barcoded reads. The sequencing reaction proceeded for between 4 and 24 hours at which point the flowcell was washed using the provided Wash Buffer. A subsequent library was loaded onto the flowcell thereafter; a maximum of three libraries were sequenced on each flowcell. Raw reads were obtained as fast5 files and subsequently analyzed. An identical procedure was followed for an R9 MinION flowcell prior to the R9.4 SpotON iteration becoming available.

### Sequencing data analysis

The bioinformatics pipeline is summarized in Fig 1B. Raw signals in fast5 files were basecalled using Oxford Nanopore Technologies Albacore command line tool (v1.2.1). Quality assessment of read data and conversion to fasta format was performed using *poretools* [58]. Best quality reads were demulitplexed, their barcode and adaptors trimmed using *porechop* [59] with a lenient barcode cut-off of 0.60, and filtered to remove reads less than 100bp and greater than 1,500bp using cutadapt 1.91 [60]. The reads were binned based on the location from which the DNA sample was obtained. Reads statistics for each run were obtained using *Nanostat* and *NanoPlot* [61].

Assigning taxonomy to each individual reads was done using QIIME 1.9.2 [62] using the Ribosomal Database Project (RDP) classifier (using Naive Bayes classification) [63]. Assignment for 16S amplified reads was done using the RDP classifier against Greengenes 13.8 rRNA reference database [64]. Assigned numbers of reads for each barcode were then compared to the total number of reads from the same barcode and multiplied by a factor of 1,000. This yields the number of reads for a given taxon per 1,000 reads. Normalization allows the comparison between samples and locations to accurately determine the abundance of a given taxon in a specific location at a specific time relative to the entire microbial community of that location or time point [65].

Quality of the read was assessed by aligning *Lambda phage* internal controls reads to a *Lambda phage* genome reference sequence and calculating percentage identity and coverage. BWA MEM v.189 [66] was used to align the reads to the reference genome with–*x ont2d* option. The outputs were converted to sam files and processed with *samtools* [67]. A similar approach was followed for processing the *Pseudomonas aeruginosa* internal control reads. Taxonomic assignment was done using the RDP classifier [63] against Greengenes 13.8 rRNA reference database [64].

### Phylogeny generation

Phylogenetic trees were obtained using assigned taxa and the PhyloT online program to generate trees in Newick format. The Newick trees were then uploaded to iTOL (Interactive Tree of Life) and phylogenies were generated using phylogenetic information and molecular data from NCBI taxonomic databases for organisms obtained in sequencing runs over 3 sampling weeks.

## Results

### Nanopore MinION DNA sequencing workflow for ambulance microbiome analysis

A total of five Nanopore DNA sequencing runs were performed. Run 1 and Run 2 included Week 1 samples taken from Ambulance B; the same set of prepared samples was used for both sequencing runs to assess reproducibility. Run 3 included the remaining samples from Week 1 (Ambulance A and C), while Run 4 and 5 included all samples (Ambulance A, B and C) from Week 2 and Week 3, respectively. The raw reads generated for the five runs ranged from 1,369 to 79,044 reads and consisted of one dimensional (1D) forward, 1D reverse and 2D reads (Table 1). The observed variation in read count may result from error associated with sample preparation and from the varied flow-cell chemistry available for each of the sequencing reactions. To sort high quality reads from the raw data, the read length was set to range from 100 bp [45] to 1,500 bp (the expected amplicon size is approximately 750 bp) with an average quality score cut-off of 10 along the sequence [45], reducing the range in read count to 538 to 57,589 reads (Table 2). This polishing step is used to ensure the presence of enough sequence similarity between the reads generated and a reference genome for accurate matching of the 16S rRNA gene. The mean read length of the filtered reads ranged from 499 bp to 819 bp (S1 Fig). The longer reads that were removed during the polishing step were likely a result of adapter ligation, and corresponding generation of long, chimeric reads [45]. The mean quality score of the filtered reads range from 10.4 to 12.8 (S1 Fig), indicating that the error rate of our sequencing is approximately 1 in 10 bases (10%) as expected [45, 68].

**Table 1:** Raw Nanopore sequencing read statistics.

Run	Week	Ambulance	Read Count	Length (bp)			Qscore		
				Mean	Min	Max	Mean	Min	Max
1	1	B	79044	724	5	69289	12.7	10	30
2	1	B	46766	1287	5	133888	11.8	10	30
3	1	A,C	1369	1405	5	77362	10.5	10	20.9
4	2	A,B,C	13585	2087	5	174586	13	10	30
5	3	A,B,C	1626	2042	5	104662	11.7	10	17.8

Read length and quality scores of the Nanopore sequencing data runs.

**Table 2:** Filtered Nanopore sequencing read statistics.

Run	Week	Ambulance	Read Count	Length (bp)			Qscore		
				Mean	Min	Max	Mean	Min	Max
1	1	B	57589	638	100	1500	12.8	10.0	18.3
2	1	B	19707	786	100	1500	11.7	10.0	16.9
3	1	A,C	558	499	100	1493	10.4	10.0	15.2
4	2	A,B,C	2493	819	100	1500	12.3	10.0	17.6
5	3	A,B,C	538	668	100	1438	11.4	10.0	17.8

Read length and quality scores of the Nanopore sequencing data runs after filtering.

Nanopore sequencing accuracy was assessed by read coverage of internal control DNA (DNA CS) mapped to the reference *Lambda phage* sequence using BWA MEM v.189 [66] (S2 Fig). Sequencing accuracy is defined as the number of matched nucleotides divided by the total number of matches and mismatches. DNA CS was added to the sampling control prior to barcoding (barcoded DNA CS), as well as to the pooled barcoded samples (unbarcoded DNA CS). Both the barcoded and unbarcoded internal control DNA achieved a sequencing accuracy greater than 71% with a mean accuracy of 85%. This is comparable to previous studies reporting accuracies in the range of 65–88% [49, 68–70]. The mean sequencing accuracy was similar for barcoded (85%) and unbarcoded (85%) samples, indicating both provided reliable sequencing information. To further validate the accuracy of 16S rRNA gene amplification, a second internal control, also representing a commonly found HAI, containing *Pseudomonas aeruginosa* genomic DNA was amplified, barcoded and sequenced for each run (S3 Fig). Greater than 65% of reads mapped to the *Pseudomonas aeruginosa* 16S rRNA gene with a mean accuracy of 83%, providing confidence in the amplification accuracy.

### Ambulance microbial diversity

For Week 1, approximately 38% of the filtered reads were assigned to the order and family classification, 5% to genus and 3% to species. For Week 2 and 3, a greater number of filtered reads were assigned to each taxonomy with 65% of the reads from Week 2 and 76% of the reads from Week 3 assigned to the order level, and 55% of the reads from Week 2 and 72% of the reads from Week 3 assigned to the family level. Approximately 25% of the reads from Week 2 and 3 were assigned to the genus level and 20% assigned to the species level. Of the total filtered reads, 90.1% were classified as bacteria, 0.7% as archaea and 9.2% as unclassified, with the latter referring to sequences that were not mapped to any reference genome.

A total of thirteen bacterial genera containing important pathogenic species were detected in ambulances (Table 3). Of particular interest *Clostridium spp*. and *Staphylococcus spp*. were detected, which are the leading cause of HAIs [71]. Other notable bacterial genera include: *Campylobacter*, the leading cause of bacterial gastroenteritis in the developed world [72]; *Listeria*, which results in a fatal bacterial illness [73, 74]; and *Shigella*, a highly contagious bacteria that causes the infectious disease shigellosis [75, 76].

**Table 3:** Opportunistic pathogenic bacteria identified in EMS vehicles at the genus level.

Genus	Pathogenic Species	Membrane	Clinical Relevance	References
*Actinobacillus*	*A*. *actinomycetemcomitans*, *A*. *urea*, *A*. *hominis*	Gram-negative	- Referred to as an oral opportunistic pathogen *Associated Disease*: Periodontitis; endocarditis; respiratory tract infections.	[77]
*Campylobacter*	*C*. *jejuni*, *C*. *coli*	Gram-negative	- Common cause of bacterial gastro-enteritis - Known to cause death in immunocompromised patients *Associated Disease*: Enteritis.	[72]
*Capnocytophaga*	*C*. *bacteremia*	Gram-negative	- Common cause of infection for immunocompromised patients with granulocytopenia and oral ulcerations *Associated Disease*: Bacteremia; endocarditis; pericardial abscess; lung infections; bone infections.	[78]
*Clostridium*	*C*. *difficile*, *C botulinum*, *C perfringens*, *C*. *tetani*,	Gram-positive	- Commonly associated with HAIs *Associated Disease*: Enterocolitis; botulism; diarrhea; tetanus.	[79]
*Haemophilus*	*H*. *influenza*, *H*. *ducreyi*,	Gram-negative	- Commonly associated with HAIs *Associated Disease*: Respiratory infections; chancroid; sepsis and bacterial meningitis in young children.	[80]
*Listeria*	*L*. *monocytogenes*	Gram-positive	- Listeriosis has a 30% mortality rate *Associated Disease*: Listeriosis, sepsis; meningitis; encephalitis; abscess formation.	[73, 74, 81]
*Pasteurella*	*P*. *multocida*	Gram-negative	- Classified as a zoonotic pathogen. *Associated Disease*: Pneumonia; chronic abscesses; edema; fibrosis.	[82]
*Prevotella*	*P*. *dentalis*, *P*. *melaninogenica*	Gram-negative	*Associated Disease*: Abscess formation; wound infection; genital tract infection; necrosis; oral and dental infection.	[83]
*Roseomonas*	*R*. *mucosa*, *R*. *gilardii*, *R*. *cervicalis*	Gram-negative	-Typically associated with the human microbiota but has been emerging as an opportunistic pathogen *Associated Disease*: Bacteremia.	[84]
*Shigella*	*S*. *dysenteriae*, *S*. *flexneri*, *S*. *boydii*, *S*. *sonnei*	Gram-negative	*Associated Disease*: Diarrhea; dysentery; inflammation of the colon and hemolytic uremic syndrome.	[75, 76]
*Staphylococcus*	*S*. *aureus*, *S*. *epidermidis*, *S*. *saprophiticus*	Gram-positive	- Multi-drug resistant pathogen - Commonly associated with HAIs *Associated Disease*: Osteomyelitis; endocarditis; surgical wound infection; urinary tract infections.	[85, 86]
*Stenotrophomonas*	*S*.*maltophilia*	Gram-negative	- Multi-drug resistant pathogen. - Causes infection in cancer patients *Associated Disease*: Respiratory tract infections; bacteremia; sepsis; endocarditis; meningitis.	[87]
*Streptococcus*	*S*.*pyogenes*, *S*. *pneumoniae*, *S*. *dysgalactiae*, *S*. *bovis*	Gram-positive	- Commonly associated with HAIs - *S*. *pneumoniae* is the primary agent responsible for pneumonia *Associated Disease*: Pharyngitis; scarlet fever; necrotizing fasciitis, myositis, toxic shock syndrome; meningitis; neonatal sepsis; pneumonia; urinary tract infection; skin infection; endocarditis.	[88, 89]

**Fig 2:**
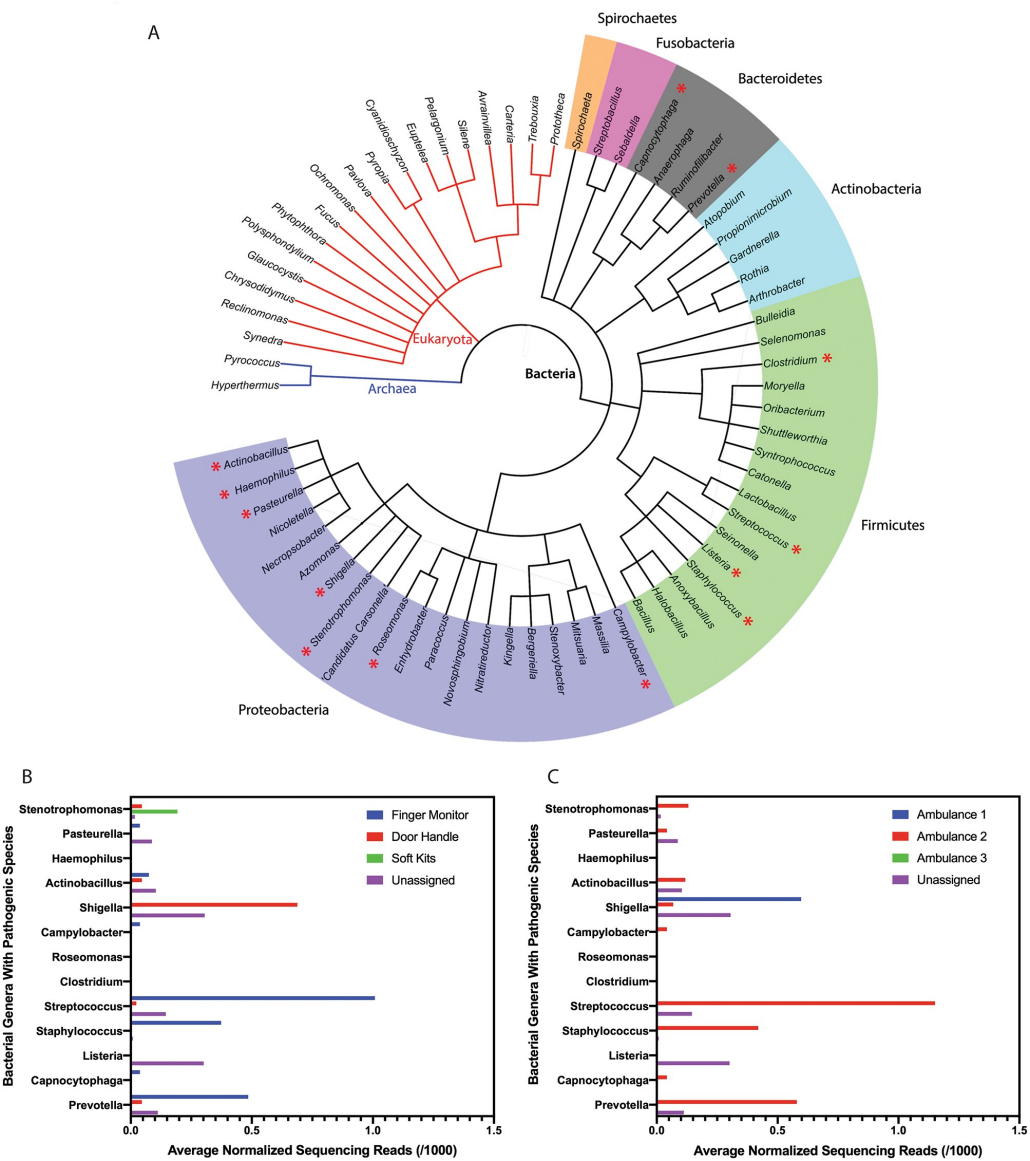
Diversity of bacterial genera detected in ambulances. (A) Phylogenetic analysis of ambulance microbiota with confidence to the genus level. Colored segments correspond to unique phyla of bacteria. Organisms are unique to the EMS vehicles and were not identified in the control samples. Pathogenic genera are indicated by a red asterisk. (B) Relative abundance of bacterial genera with pathogenic species, represented as the average normalized sequencing reads detected over a three-week period from all ambulances for each sampling location and (C) from all sampling locations for each ambulance.

A phylogenetic tree was generated using the online program PhyloT (Fig 2A). Sixty-eight unique organisms were identified at the genus level: two classified as Archaea, eighteen classified as Eukaryota, and forty-eight classified as Bacteria. The bacterial genera are further classified into six different phyla: Spirochaetes, Fusobacteria, Bacteroidetes, Actinobacteria, Firmicutes, and Proteobacteria. These different phyla were not found in the control sample and represent the diverse population of microorganisms present and that can be detected in EMS vehicles. The phylogenetic tree illustrates the range of potential genera within an ambulance at any given time and highlights that several of these genera contain opportunistic pathogenic organisms, including *Actinobacillus*, *Campylobacter*, *Capnocytophaga*, *Clostridium*, *Haemophilus*, *Listeria*, *Pasteurella*, *Prevotella*, *Roseomonas*, *Shigella*, *Staphylococcus*, *Stenotrophomonas*, and *Streptococcus* (indicated by the red star). Approximately 4% of the reads assigned to the genera level are bacteria with pathogenic species. Although representing a relatively minor component of the sequencing reads, the identification of any bacteria with pathogenic species is an important consideration when transporting and treating immunocompromised patients.

The corresponding bar graph shows the variation in relative abundance of bacterial genera with pathogenic species detected at each sampling location (Fig 2B) and in each ambulance (Fig 2C), including sequencing reads that were not assigned to any location or ambulance. A complete summary highlighting the variation in bacterial genera detected for all bacterial genera is included in S4 Fig. While several of these genera are also members of the human microbiome [90], it is worth noting that representative species from these genera are capable of causing illness, and are of concern to both medical professionals and immunocompromised individuals as they pose a potential threat to the welfare of patients and paramedics within ambulances.

Our analysis identified a “yet-to-be-cultured” genera: “*Candidatus Carsonella”*. This organism is given the “*Candidatus”* designation since it has not been cultured in the laboratory, and thus would not have been detected using traditional cell-culturing methods [91]. We detected non-harmful, commensal bacteria as expected such as *Lactobacillus*, common to the human microbiome, as well as *Bacillus*, common to the urban built environment [92]. Several eukaryotic mitochondria were also identified by our approach, and assignment of these organisms was made confidently due to the presence of 16S rRNA genes in the mitochondrial genome.

**Fig 3:**
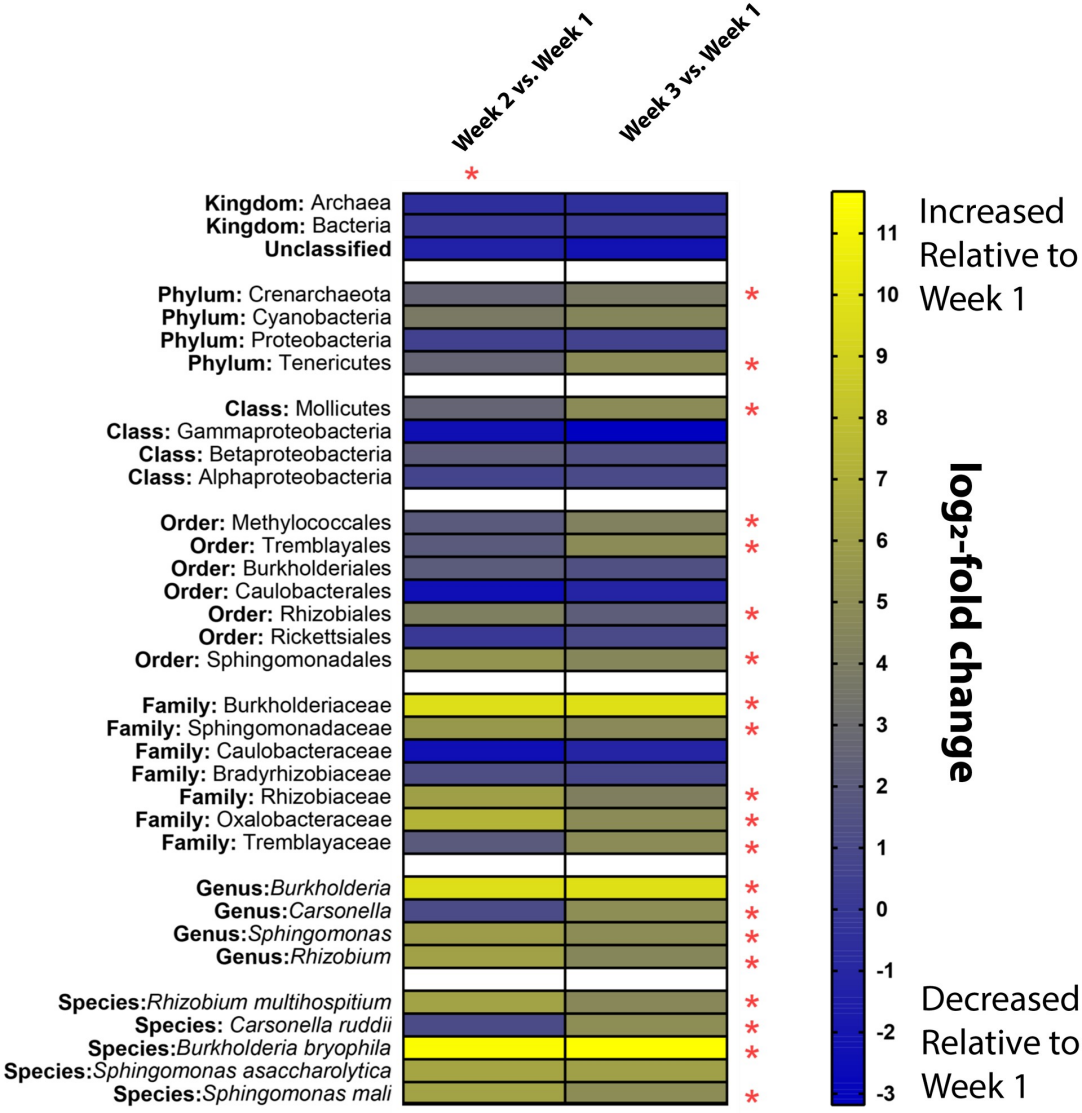
A comparison of fold-change of taxa found in ambulances relative to first sampling event. Heatmap illustrating the log_2_-fold change of organisms sequenced using ONT MinION relative to the first week of sampling. Yellow squares correspond to an increase in a given taxa at a corresponding week relative to Week 1, while blue squares indicate a decrease in abundance of a taxa relative to Week 1. Asterisks indicate a significant change relative to Week 1 (p<0.05). All changes from week 2 are significant relative to week 1 as indicated by the red asterisk.

The temporal change in relative abundance of the microbial composition in relation to Week 1 is shown using heat maps at each taxonomic level (Fig 3). The heat maps illustrate the log_2_-fold change in each taxonomic unit from Week 2 and 3 compared to Week 1 samples. A z-score was calculated by taking the difference between the total normalized reads assigned to a given taxon at Week 2 or 3 and Week 1 and dividing it by the square root of the sum of the reads assigned to a given taxon for the same weeks. A *p* value is then calculated from the assigned z-score, and if <0.05, the difference is considered significant [93]. This allows for monitoring of enrichment of certain species over time such as *Burkholderia*, which had a fold change increase of 9.72 and 9.87 in Week 2 and 3 relative to Week 1. For other microorganisms, their abundance decreased over time, such as members of the family Caulobacteraceae, which had a fold change decrease of 2.36 and 1.13 in Week 2 and 3 relative to Week 1. This technique provides a tool for monitoring the dynamics of a microbiome over time, allowing for trends to be monitored and recorded. Ultimately, this enables a better understanding of pathogen transmission, cleaning effectiveness and identifying how the microbiome composition contributes to microbial spread.

## Discussion

### MinION sequencing platform

For rapid identification of the microbial community, the MinION was used, which is advantageous due to its small size, portability, and rapidly developing sequencing technology. Sample preparation and sequencing using the MinION is accelerated with each iteration of the platform. For example, recently developed kits and devices, such as the ONT VolTRAX and SmidgION, simplify and speed up sample preparation, barcoding, and analysis, further highlighting the potential for on-site sequencing of EMS vehicles. Recently commercialized flow cells can now process up to 450 bases of DNA per second and per channel, allowing for fast data acquisition. Additionally, the sequencing platform is scalable with use of the PromethION benchtop sequencer which allows for many flow cells to be run in parallel yielding an increasingly more comprehensive picture of the microbial community. Studies have also shown the use of ONT’s sequencing devices to identify antibiotic resistance genes, as well as specific viruses, by using alternative primers during amplification showing the diverse types of organisms that can be identified using this platform [28, 49].

While the sequencing approach used is indeed streamlined and portable, there are elements of the pipeline that could be improved. The first of these concerns is the number of very long (>5,000bp) reads that were obtained from our sequencing runs, which were filtered out. These are likely a result of improper adapter ligation and creation of chimeric species of the amplified PCR products. This type of ligation artifact has been improved in current iterations of ONT sequencing kits using ligase free adapter addition (16S Barcoding Kit SQk-RAB204). Second, although our approach was reproducible, as results obtained in our first run were consistent with the results of our second run using newly prepared samples, subsequent runs using the same flow cell exhibited a reduced read number. This indicates a limitation to how many times a flow cell can be re-used. The rapid development of the ONT system also necessitates flexibility in sample preparation and sequencing. As the sequencing runs are often carried out on separate flow cells that may not behave in the same way, further optimization of the reproducibility and quality of reads between flow cells should be considered. The Flongle, a new device from ONT ideal for small-scale, rapid tests, provides real-time sequencing information and can be used to assess the quality of a run before performing a large-scale experiment. This device provides a promising approach for improving read quality and for real-time, on-site assessment.

### Towards deciphering the ambulance microbiome

Analysis of the sequencing data resulted in the identification of sixty-eight unique organisms at the genus level. Our approach provided a new and more comprehensive picture of the microbial community in ambulances in comparison to cell-culturing techniques used in previous studies, which focused on the identification of specific pathogens of interest, such as MRSA [15, 17–19, 21, 22]. This is a result of the ability to identify any organism via DNA sequencing while cell-culturing techniques are limited by the need to know precisely what to look for, as well as to know the correct culturing conditions at which the bacteria will grow. For example, this work identified “*Candidatus Carsonella”*, which has not been cultured further demonstrating our ability to detect novel bacteria. 16S rRNA genes were chosen for bacterial identification due to their essentiality in prokaryotes and common use in taxonomic identification [94, 95]. These genes are also present in mitochondrial genomes explaining why a proportion of our reads are classified as Eukaryotes in the phylogenetic tree [96]. Regarding the identification of organisms associated with HAIs [71], we detected three bacterial genera that were also reported in the metagenomics analysis of ambulances in the United States; *Haemophilis*, *Staphylococcus*, *and Stenotrophomonas* [27]. Additionally, we detected *Clostridium* and *Streptococcus*, two genera commonly associated with HAIs, that were not reported in the US based study.

The bacterial community was shown to vary both between locations and over time. Potential pathogenic bacteria were located primarily on the finger monitor in direct contact with patients, secondarily on the door handle in direct contact with the EMS workers, and also identified on the soft kits in contact with the environment. Differences were also observed when comparing between ambulances and, in comparison to Week 1, select organisms were decreased or enriched, suggesting that the cleaning products and cycle used were effective for some bacteria but not all, and that more stringent disinfection might be required. The observed differences in the microbial community may also be impacted by the frequency each vehicle and piece of equipment is used, as well as the type of emergency responded to, which would vary from week to week.

While it is likely not possible to ensure a workspace is sterile for the duration of the workday, our work shows that methods to monitor vehicle cleanliness can maintain proper hygiene. Building on the described approach, emergency medical personnel will be able to rapidly analyze the microbial load in less than 24 hours and modify their cleaning practices as necessary. This will reduce unnecessary cleaning and costs, while in general making targeted cleaning much easier. Additionally, identification of select species within a few minutes can be introduced using paper-based tests designed to detect pathogens of interest previously identified in the sequencing runs [97]. Emerging technology from ONT, such as the VolTRAX, Flongle and SmidgION, will increase the accessibility of microbiome analysis by providing simple, automated and easy to use sequencing devices that do not require a strong scientific background for operation.

### Limitations

This proof of principle study successfully identified variations in the EMS microbiome and the presence of bacterial genera that contain important pathogenic species. As a result of re-using flow cells, leading to a low sequencing read count, the identification of bacteria was limited to the genus level. Sequencing is expected to improve with the availability of new flow cell chemistry continually being upgraded to provide better basecalling accuracy. Maximizing the amount of DNA extracted will further improve the accuracy of identifying and detecting bacteria present. Previous work using a mock microbial community achieved species-level resolution with the MinION [43], suggesting increasing coverage of the 16S rRNA gene improves read assignment. Further optimization is required to achieve this resolution when processing environmental samples from EMS vehicles, which contain dirt and other material not present in mock microbial communities.

By amplification of the 16S rRNA gene, we restricted identification in this study to bacteria excluding the identification of viruses or fungi. To broaden the detection, less biased methods such as Multiple Displacement Amplification (MDA) [55] to amplify whole genomes or the use of additional primer pairs designed to identify fungi or viruses of interest can be integrated [98, 99]. There is also the option to include a pre-treatment step using chemical agents, such as propidium monoazide, which prevents DNA from dead cells to be amplified, ensuring amplification of DNA from viable bacteria [100]. To be accessible by the EMS, the described pipeline uses basic molecular biology tools and equipment. Including additional steps, such as MDA or propidium monoazide treatment, can expand and improve the sequencing results.

Other efforts to streamline our pipeline include implementation of new kits and devices available from ONT (such as “The Rapid Sequencing Kit” and the VolTRAX device), which would allow for simplified sample acquisition, preparation and sequencing. On this background, the described MinION sequencing pipeline provides a tool to detect variations in the EMS microbiome, which has great utility for assessing disease transmission and maintenance within EMS. Improvements addressing the respective design limitations will further extend the utility of the reported approach.

## Conclusion

Our pipeline provides a detection and monitoring platform giving EMS personnel a means of rapid feedback and evaluation of best cleaning practices within ambulances, and is a step towards understanding the ambulance microbiome. Through our initial study, we demonstrate the ability to identify potential hot spots that require more attention and monitoring throughout cleaning cycles, such as the SPO_2_ finger monitor. For the three locations sampled, we also provided our local EMS with the necessary data to identify cleaning products that effectively eliminate the pathogens detected. Since many of the potentially pathogenic bacteria were found on the finger monitor, an area of high contact by patients during transport and a difficult area to clean, there is a high likelihood that ambulances are indeed vehicles for pathogens into hospitals and *vice versa*. We recommend that a more detailed analysis and tracking of pathogen spread between these environments should be implemented to mitigate this potential health hazard. Large-scale analysis of the sequencing data generated over a longer period will also enable epidemiological studies and the identification of new emerging pathogens, improving our ability to predict and mitigate pathogen outbreaks.

## Supporting information

S1 Fig Quality score and read length distribution of MinION DNA sequencing reads. (A) Run 1. (B) Run 2. (C) Run 3. (D) Run 4. (E) Run 5.
(PDF)

S2 Fig Read Coverage of the internal control DNA CS mapped to the *Lambda phage* genome. (A) Run 1 barcoded. (B) Run 2 barcoded. (C) Run 4 barcoded. (D) Run 1 unbarcoded. (E) Run 2 unbarcoded. (F) Run 4 unbarcoded.
(PDF)

S3 Fig Read coverage of *Pseudomonas aeruginosa* mapped to the reference genome. (A) Run 1 barcoded. (B) Run 2 barcoded. (C) Run 4 barcoded. (D) Run 1 unbarcoded. (E) Run 2 unbarcoded. (F) Run 4 unbarcoded.
(PDF)

S4 Fig Relative abundance of bacterial genera. Represented as the average normalized sequencing reads for bacterial genera detected over a three-week period from (A) all ambulances for each sampling location and (B) from all sampling locations for each ambulance. (C) Average normalized sequencing reads for bacterial genera detected in all three ambulances and sampling locations for each week.
(PDF)
