# Supplementary material for: Rapid metagenomics analysis of EMS vehicles for monitoring pathogen load using nanopore DNA sequencing
Source: PLoS One. 2019 Jul 24;14(7):e0219961. doi: 10.1371/journal.pone.0219961 (PMC6655686; doi:10.1371/journal.pone.0219961)
Supplement: S1 Fig — (A) Run 1. (B) Run 2. (C) Run 3. (D) Run 4. (E) Run 5. (PDF) [file pone.0219961.s001.pdf]

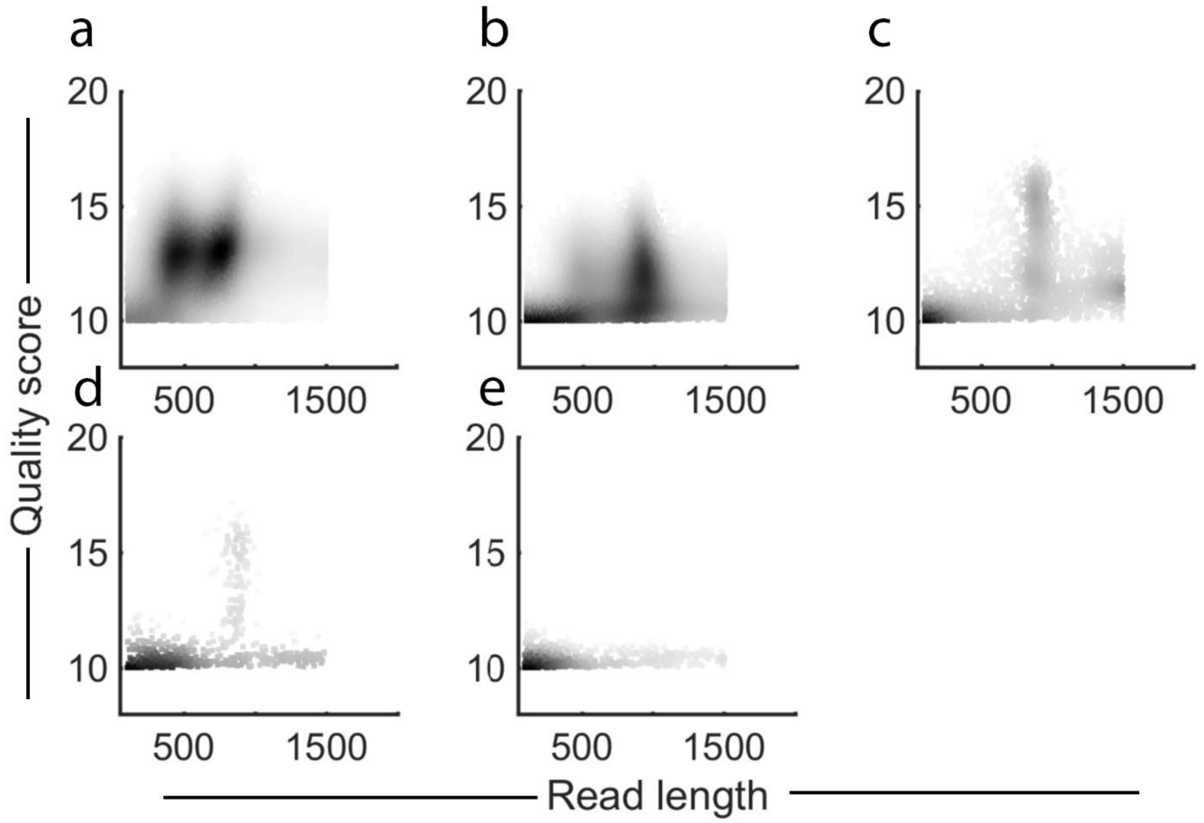

S1 Fig. Quality score and read length distribution of MinION® DNA sequencing reads. (A) Run 1. (B) Run 2. (C) Run 3. (D) Run 4. (E) Run 5.
