# Supplementary material for: Rapid metagenomics analysis of EMS vehicles for monitoring pathogen load using nanopore DNA sequencing
Source: PLoS One. 2019 Jul 24;14(7):e0219961. doi: 10.1371/journal.pone.0219961 (PMC6655686; doi:10.1371/journal.pone.0219961)
Supplement: S3 Fig — (A) Run 1 barcoded. (B) Run 2 barcoded. (C) Run 4 barcoded. (D) Run 1 unbarcoded. (E) Run 2 unbarcoded. (F) Run 4 unbarcoded. (PDF) [file pone.0219961.s003.pdf]

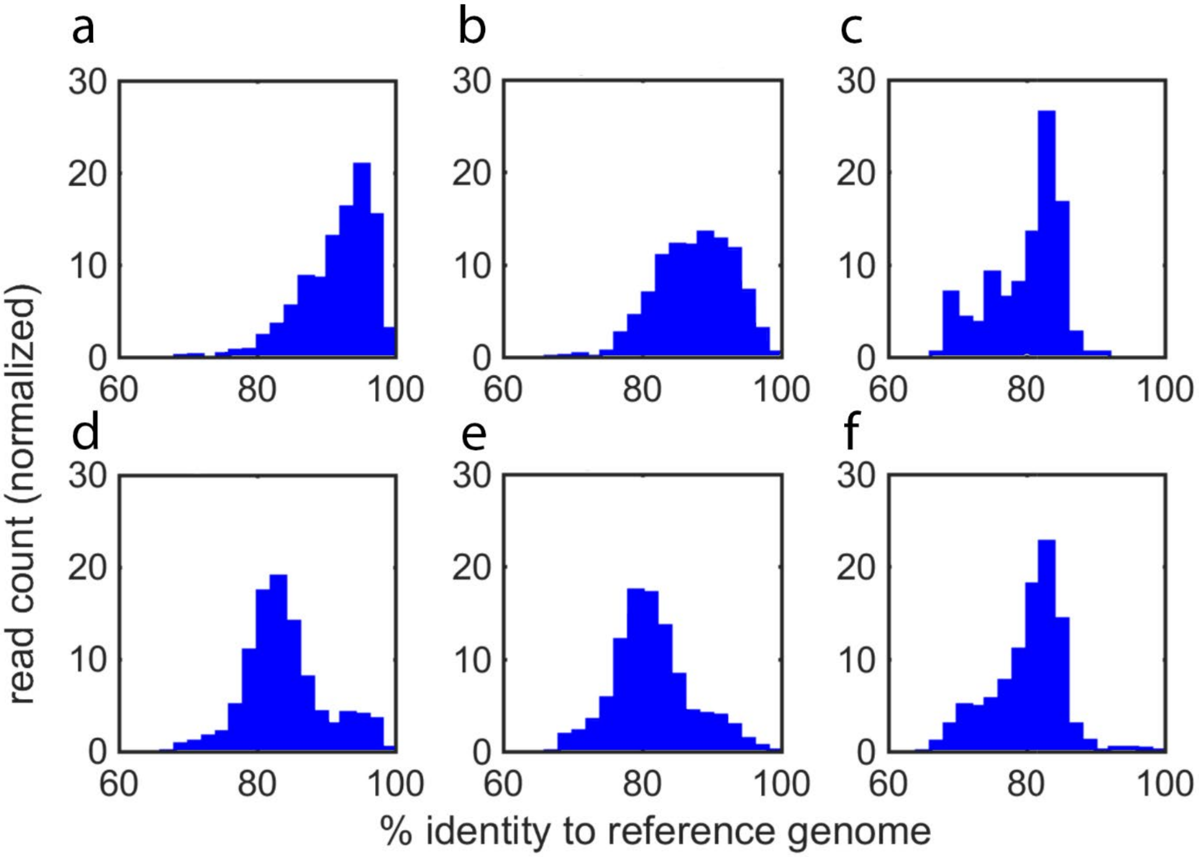

S3 Fig. Read coverage of *Pseudomonas aeruginosa* mapped to the reference genome. (A) Run 1 barcoded. (B) Run 2 barcoded. (C) Run 4 barcoded. (D) Run 1 unbarcoded. (E) Run 2 unbarcoded. (F) Run 4 unbarcoded.
