# Supplementary material for: Rapid metagenomics analysis of EMS vehicles for monitoring pathogen load using nanopore DNA sequencing
Source: PLoS One. 2019 Jul 24;14(7):e0219961. doi: 10.1371/journal.pone.0219961 (PMC6655686; doi:10.1371/journal.pone.0219961)
Supplement: S4 Fig — Represented as the average normalized sequencing reads for bacterial genera detected over a three-week period from (A) all ambulances for each sampling location and (B) from all sampling locations for each ambulance. (C) Average normalized sequencing reads for bacterial genera detected in all three ambulances and sampling locations for each week. (PDF) [file pone.0219961.s004.pdf]

A

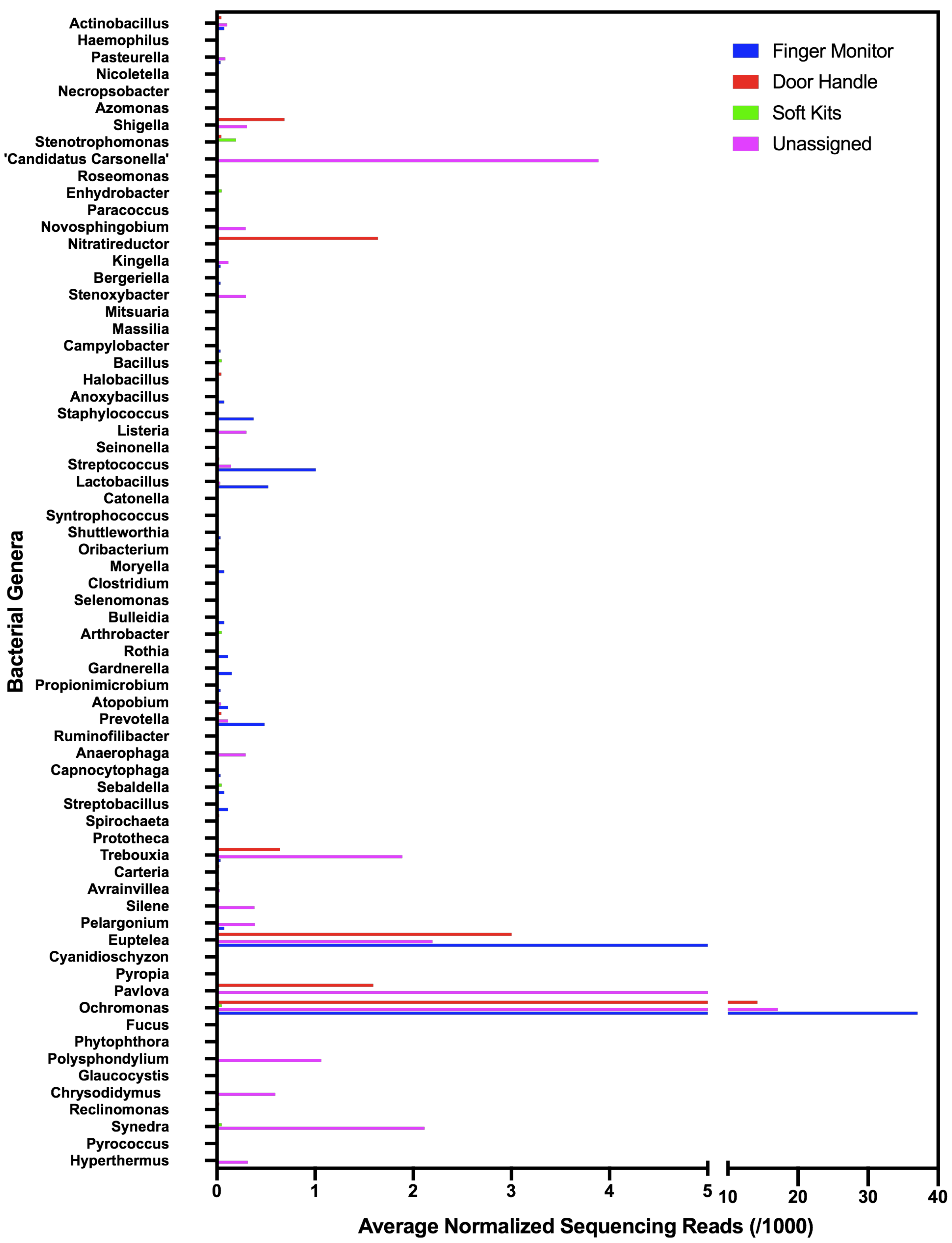

Finger Monitor
Door Handle
Soft Kits
Unassigned
Bacterial Genera
Actinobacillus
Haemophilus
Pasteurella
Nicoletella
Necropsobacter
Azomonas
Shigella
Stenotrophomonas
'Candidatus Carsonella'
Roseomonas
Enhydrobacter
Paracoccus
Novosphingobium
Nitratireductor
Kingella
Bergeriella
Stenoxybacter
Mitsuaria
Massilia
Campylobacter
Bacillus
Halobacillus
Anoxybacillus
Staphylococcus
Listeria
Seinonella
Streptococcus
Lactobacillus
Catonella
Syntrophococcus
Shuttleworthia
Oribacterium
Moryella
Clostridium
Selenomonas
Bulleidia
Arthrobacter
Rothia
Gardnerella
Propionimicrobium
Atopobium
Prevotella
Ruminofilibacter
Anaerophaga
Capnocytophaga
Sebaldella
Streptobacillus
Spirochaeta
Prototheca
Trebouxia
Carteria
Avrainvillea
Silene
Pelargonium
Euptelea
Cyanidioschyzon
Pyropia
Pavlova
Ochromonas
Fucus
Phytophthora
Polysphondylium
Glaucocystis
Chrysodidymus
Reclinomonas
Synedra
Pyrococcus
Hyperthermus
0
1
2
3
4
5
10
20
30
40
Average Normalized Sequencing Reads (/1000)

B

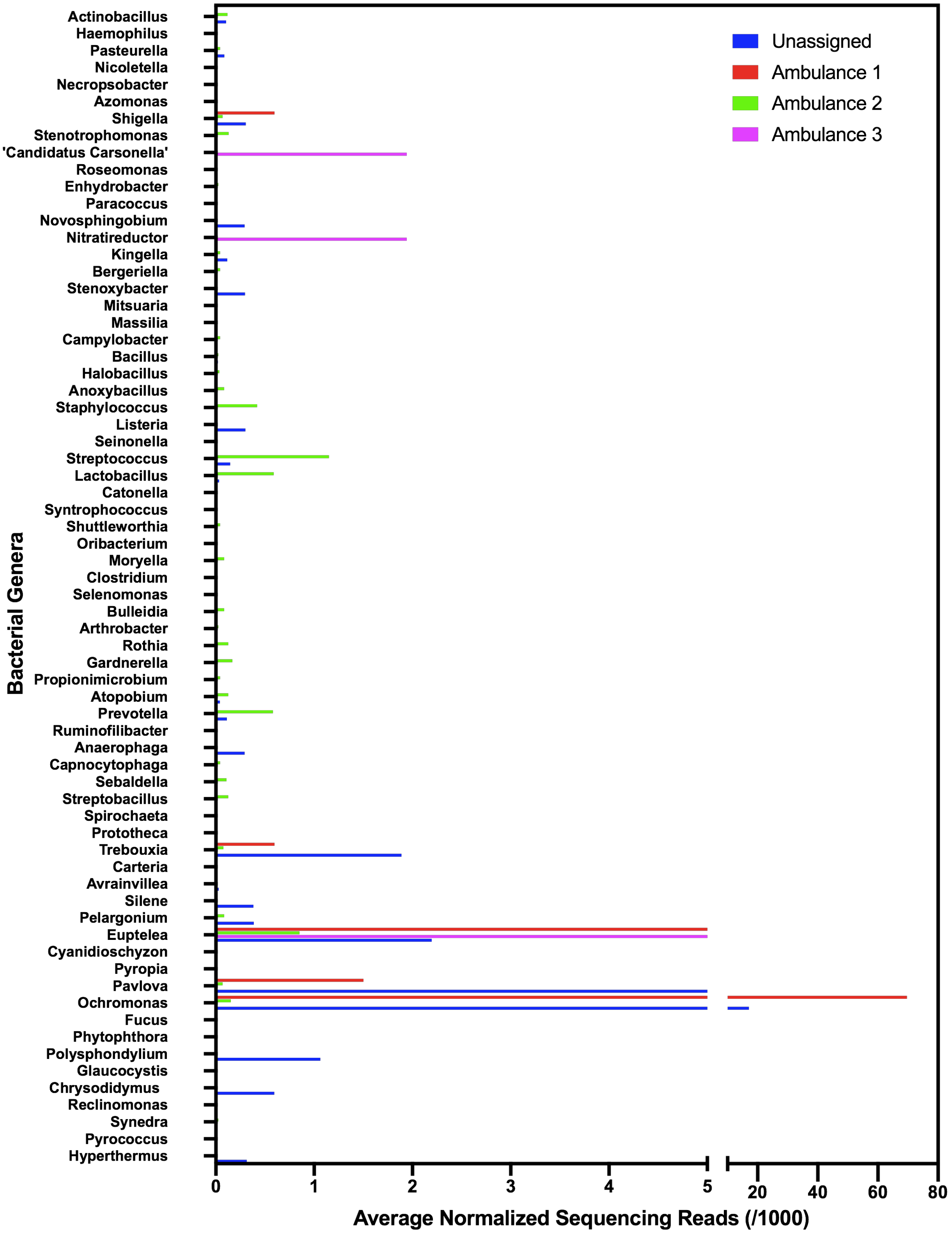

Unassigned
Ambulance 1
Ambulance 2
Ambulance 3
Bacterial Genera
Actinobacillus
Haemophilus
Pasteurella
Nicoletella
Necropsobacter
Azomonas
Shigella
Stenotrophomonas
'Candidatus Carsonella'
Roseomonas
Enhydrobacter
Paracoccus
Novosphingobium
Nitratireductor
Kingella
Bergeriella
Stenoxybacter
Mitsuaria
Massilia
Campylobacter
Bacillus
Halobacillus
Anoxybacillus
Staphylococcus
Listeria
Seinonella
Streptococcus
Lactobacillus
Catonella
Syntrophococcus
Shuttleworthia
Oribacterium
Moryella
Clostridium
Selenomonas
Bulleidia
Arthrobacter
Rothia
Gardnerella
Propionimicrobium
Atopobium
Prevotella
Ruminofilibacter
Anaerophaga
Capnocytophaga
Sebaldella
Streptobacillus
Spirochaeta
Prototheca
Trebouxia
Carteria
Avrainvillea
Silene
Pelargonium
Euptelea
Cyanidioschyzon
Pyropia
Pavlova
Ochromonas
Fucus
Phytophthora
Polysphondylium
Glaucocystis
Chrysodidymus
Reclinomonas
Synedra
Pyrococcus
Hyperthermus
0
1
2
3
4
5
20
40
60
80
Average Normalized Sequencing Reads (/1000)

C

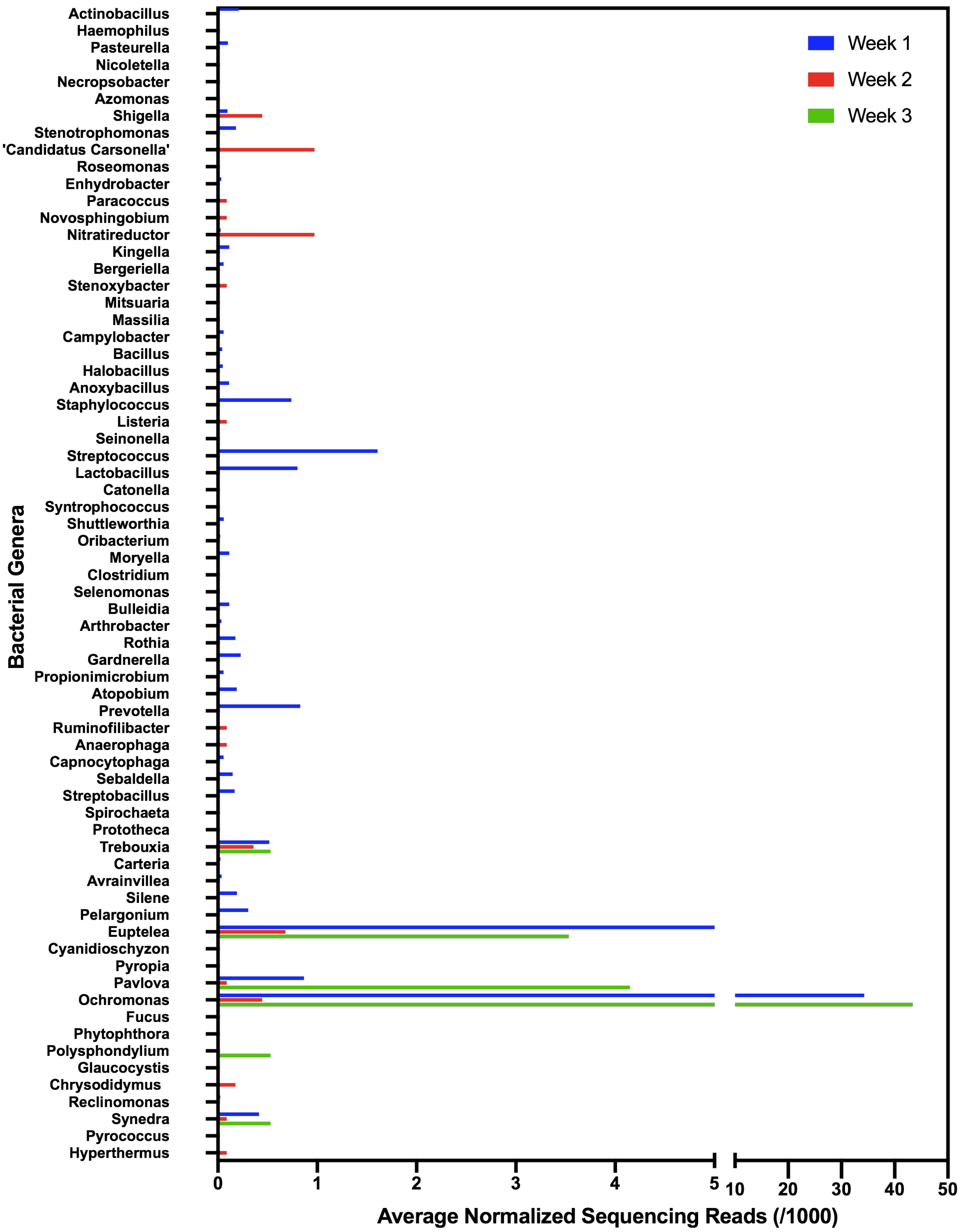

Actinobacillus
Haemophilus
Pasteurella
Nicoletella
Necropsobacter
Azomonas
Shigella
Stenotrophomonas
'Candidatus Carsonella'
Roseomonas
Enhydrobacter
Paracoccus
Novosphingobium
Nitratireductor
Kingella
Bergeriella
Stenoxybacter
Mitsuaria
Massilia
Campylobacter
Bacillus
Halobacillus
Anoxybacillus
Staphylococcus
Listeria
Seinonella
Streptococcus
Lactobacillus
Catonella
Syntrophococcus
Shuttleworthia
Oribacterium
Moryella
Clostridium
Selenomonas
Bulleidia
Arthrobacter
Rothia
Gardnerella
Propionimicrobium
Atopobium
Prevotella
Ruminofilibacter
Anaerophaga
Capnocytophaga
Sebaldella
Streptobacillus
Spirochaeta
Prototheca
Trebouxia
Carteria
Avrainvillea
Silene
Pelargonium
Euptelea
Cyanidioschyzon
Pyropia
Pavlova
Ochromonas
Fucus
Phytophthora
Polysphondylium
Glaucocystis
Chrysodidymus
Reclinomonas
Synedra
Pyrococcus
Hyperthermus
Bacterial Genera
Week 1
Week 2
Week 3
0
1
2
3
4
5
10
20
30
40
50
Average Normalized Sequencing Reads (/1000)

**S4 Fig. Relative abundance of bacterial genera.** Represented as the average normalized sequencing reads for bacterial genera detected over a three week period from (A) all ambulances for each sampling location and (B) from all sampling locations for each ambulance. (C) Average normalized sequencing reads for bacterial genera detected in all three ambulances and sampling locations for each week.
